# Impact of prednisolone tapering and extended dupilumab dosing intervals on comorbid asthma in patients with chronic rhinosinusitis with nasal polyps

**DOI:** 10.1016/j.jacig.2025.100605

**Published:** 2025-11-14

**Authors:** Shunsuke Minagawa, Masahiro Yoshida, Daiki Nakashima, Takanori Numata, Shun Inukai, Tomoya Maruyama, Tetsuya Adachi, Jun Araya, Yoshinori Matsuwaki

**Affiliations:** a Matsuwaki Clinic Shinagawa, Tokyo, Japan; b Division of Respiratory Diseases, Department of Internal Medicine, The Jikei University School of Medicine, Tokyo, Japan; c Department of Otorhinolaryngology, The Jikei University School of Medicine, Tokyo, Japan

**Keywords:** CRSwNP, dupilumab, asthma, systemic corticosteroids, united airway disease

## Abstract

**Background:**

Chronic rhinosinusitis with nasal polyps (CRSwNP) frequently coexists with asthma and involves type 2 inflammation. Dupilumab enables prednisolone (PSL) tapering and extended dosing intervals in CRSwNP, but the impact on comorbid asthma is unclear.

**Objectives:**

We evaluated the impact of PSL tapering and extended dupilumab dosing intervals on asthma outcomes in patients with CRSwNP.

**Methods:**

We retrospectively analyzed 60 patients with CRSwNP and comorbid asthma treated with dupilumab between 2020 and 2023 who reduced oral PSL to ≤1 mg within 1 year on dupilumab administered every 2 weeks and then extended the dosing interval to ≥3 weeks. Airway outcomes including nasal polyp score, CT score, olfactory function, asthma control test, spirometry, oscillometry, fractional exhaled nitric oxide, blood eosinophils, serum IgE, and annual exacerbation frequency were assessed at baseline, 3 months, and annually up to 3 years.

**Results:**

Nasal polyp score, CT score, asthma control test and fractional exhaled nitric oxide improved within 3 months and remained stable during PSL tapering and interval extension. Forced expiratory volume in 1 second and R5 improved transiently but gradually returned toward baseline over time. Eosinophil counts rose after PSL tapering but subsequently declined despite reduced dupilumab frequency. Total IgE and exacerbation frequency remained consistently suppressed. Subgroup analyses showed that asthma control improved or remained well controlled irrespective of baseline severity or step-down in asthma treatment.

**Conclusions:**

In patients with CRSwNP and comorbid asthma, PSL tapering and extension of dupilumab dosing intervals were feasible and did not worsen asthma control when sinonasal disease remained well controlled.

Chronic rhinosinusitis with nasal polyps (CRSwNP) is a recalcitrant type 2 inflammatory disorder that is frequently comorbid with bronchial asthma. The strong association between upper and lower airway diseases has given rise to the concept of *united airway disease* (UAD),^1^^,^^2^ highlighting the shared immunopathology across both anatomical regions. Dupilumab, a monoclonal antibody targeting IL-4 and IL-13 signaling via IL-4Rα inhibition, has emerged as an effective treatment for CRSwNP and asthma, offering significant reductions in nasal polyp burden, improvements in quality of life, and better asthma control.^3^^,^^4^ Recent real-world studies have demonstrated marked improvements in sinonasal symptoms and olfactory function, particularly within the first 3 months of therapy.^5^ In our clinic, we previously demonstrated that dupilumab maintained its clinical efficacy in patients with refractory CRSwNP even after systemic corticosteroid tapering and extension of dosing intervals.^6^ However, for severe asthma, current guidelines recommend continued once every 2 weeks dosing, and real-world data on the impact of extended dosing intervals on asthma control remain limited.^7^

Although the efficacy of dupilumab has been well documented, the question of long-term treatment strategies remains unresolved. A 2-year observational study demonstrated that interval tapering to every 8 weeks maintained the clinical benefits initially achieved during the first 6 months of therapy.^8^ Similarly, a Korean real-world study showed that Sinonasal Outcome Test 22–based adjustment of interdose intervals up to 8 weeks led to sustained symptom control and high patient satisfaction, suggesting the feasibility of a tailored tapering strategy.^9^ However, these studies focused mainly on sinonasal outcomes and did not thoroughly evaluate the impact of dose tapering on lower airway disease activity. Given the immunologic overlap between CRSwNP and asthma, it is clinically important to clarify whether dupilumab tapering compromises asthma control or pulmonary function.

In this study, we retrospectively evaluated patients with CRSwNP and comorbid asthma who underwent a structured tapering protocol consisting of prednisolone (PSL) dose reduction followed by extension of the dupilumab dosing interval. Our primary objective was to assess the clinical effects of this approach on both upper and lower airway outcomes over a 3-year period. By focusing on real-world data and incorporating pulmonary function and asthma control metrics, we aimed to provide evidence for the safety and feasibility of dupilumab tapering strategies in patients with UAD.

## Methods

### Study design and participants

This retrospective single-center study was approved by the ethics review board of The Jikei University School of Medicine (approval 33-407[11032]). A total of 155 patients with CRSwNP who received dupilumab therapy at Matsuwaki Clinic Shinagawa between January 2020 and December 2023 were retrospectively reviewed through electronic medical records. All patients received dupilumab specifically for the treatment of CRSwNP, as defined by the European Position Paper on Rhinosinusitis and Nasal Polyps 2020.^5^ No patients were treated for asthma alone.

At our institution, patients were typically followed at 3-month intervals after initiating dupilumab. Once patients became accustomed to self-administration and showed no adverse events, treatment response was evaluated every 3 months. If improvement in sinonasal symptoms was confirmed, oral PSL was tapered by 1 mg increments. After successful discontinuation of PSL, the dupilumab dosing interval was extended progressively—typically to every 3 or 4 weeks—according to a standardized tapering protocol previously reported.

To be eligible for inclusion in this study, patients were required to meet both of the following criteria: (1) successful reduction of oral PSL to ≤1 mg/d within 1 year of dupilumab initiation, and (2) extension of the dupilumab dosing interval to ≥3 weeks at some point after the first year. The threshold of ≤1 mg was used instead of complete discontinuation because some patients discontinued PSL slightly beyond the 1-year mark but still within 3 months of their annual assessment. Given the uniform scheduling of clinical evaluations across patients (ie, at baseline, 3 months, and annually), such cases were considered clinically equivalent and not substantially in violation of the study design.

At our institution, the timing of clinical assessments was fixed, and outcome measures were collected at predefined intervals: baseline, 3 months, 1 year, 2 years, and 3 years. If patients extended their dupilumab dosing interval before completing 1 year of treatment, their 1-year evaluation would already reflect the combined effects of PSL tapering and interval extension, making it difficult to isolate the contribution of each intervention. Therefore, to preserve the interpretability of treatment phase–specific effects and minimize confounding, we excluded patients who initiated interval extension within the first year.

**Fig 1:**
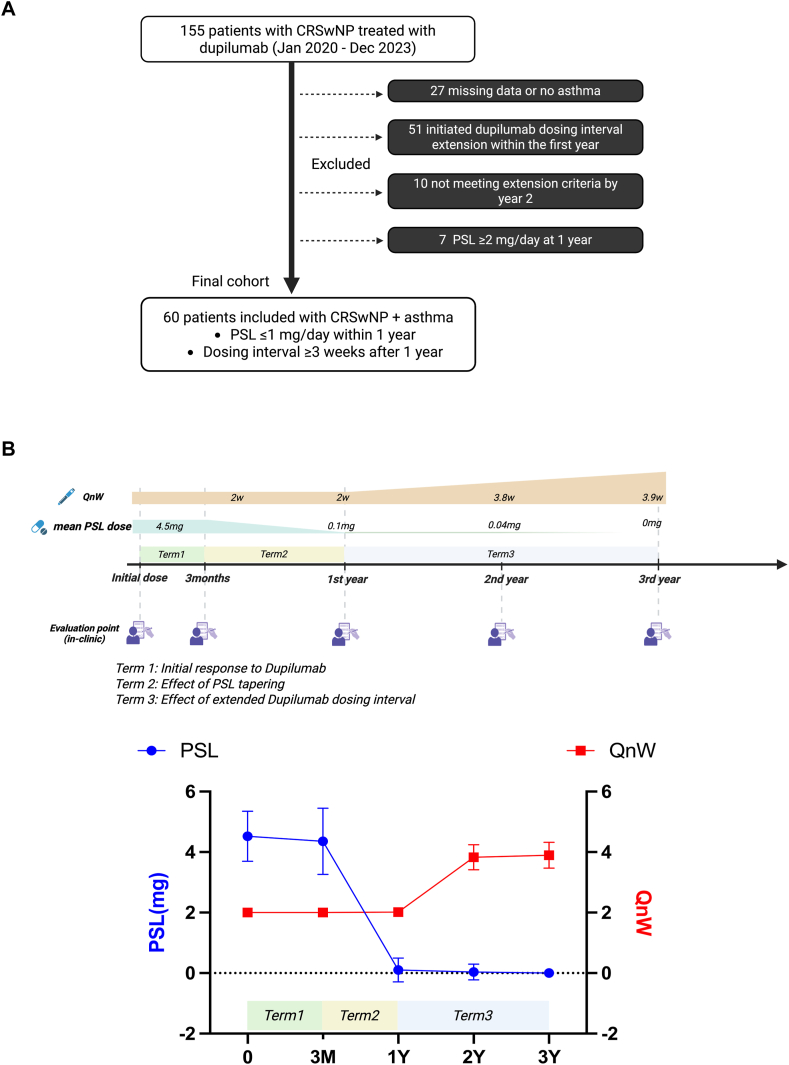
**A,** Flowchart of patient selection, inclusion, and exclusion criteria. **B,** Treatment timeline showing changes in dupilumab dosing interval (QnW), mean oral PSL dose, and scheduled clinical assessment points. *QnW* indicates dosing interval, with dose per no. of weeks (W).

Of the 155 patients reviewed, 27 were excluded because of substantial missing clinical data or lack of comorbid asthma. An additional 51 patients were excluded for having extended their dupilumab dosing interval before the 1-year time point, 4 for not meeting interval extension criteria within 2 years, and 7 for receiving PSL ≥ 2 mg/d at the 1-year visit. After these exclusions, 60 patients were included in the final analysis (Fig 1, *A*). All included patients were adults with recurrent CRSwNP after endoscopic sinus surgery, and all had comorbid bronchial asthma. Patients were excluded if they had cystic fibrosis, eosinophilic granulomatosis with polyangiitis, pregnancy, or known hypersensitivity to dupilumab.

Baseline characteristics of the study population are summarized in Table I. All 60 patients received dupilumab for the treatment of CRSwNP in accordance with the diagnostic criteria established in the European Position Paper on Rhinosinusitis and Nasal Polyps 2020.^5^ None of the patients received dupilumab for asthma alone. Similarly, all patients were receiving systemic corticosteroids (oral PSL) exclusively for the management of CRSwNP.

**Table I:** Baseline demographic and clinical characteristics of 60 patients with CRSwNP and comorbid asthma

Characteristic	Value
Age (years) at start of therapy	46.38 (10.62)
Time (days) since most recent ESS	1653 (5571)
Blood eosinophils (/μL)	351.8 (267.7)
Eosinophils (/μL) in NPs	157.8 (182.5)
Baseline NPS	5.85 (0.4444)
Asthma treatment step (GINA)	2.9 (1.285)
PSL dosage (mg)	4.525 (0.8256)
ACT	22.68 (3.825)
Annual asthma exacerbations	0.5667 (0.9088)
Feno (ppb)	56 (47.45)
FEV_1_ (L)	2.833 (0.7212)
%FEV_1_	96.33 (20.49)
R5 (cmH_2_O/L/s)	3.34 (1.239)
Fres (Hz)	8.191 (3.358)
Asthma treatment GINA step	
1	15
2	2
3	22
4	16
5	5

Data are indicated as means (SDs) or as numbers. *ESS,* Endoscopic sinus surgery.

The mean age at dupilumab initiation was 46.4 years. All patients had previously undergone endoscopic sinus surgery, with a median time of approximately 4.5 years since the last procedure. The average peripheral blood eosinophil count was 351.8 × 10^6^/L, and the mean eosinophil count in nasal polyp tissue was 157.8 × 10^6^/L, indicating a type 2 inflammatory endotype. The baseline nasal polyp score (NPS) averaged 5.85, reflecting a moderate-to-severe polyp burden. The mean daily dose of oral PSL at baseline was 4.52 mg.

All patients had comorbid bronchial asthma. Asthma control at baseline was generally favorable, with a mean Asthma Control Test (ACT) score exceeding 20, low annual exacerbation frequency, and relatively preserved lung function as measured by forced expiratory volume in 1 second (FEV_1_). Asthma treatment steps ranged from Global Initiative for Asthma (GINA) step 1 to step 5, with the disease of relatively few patients classified as step 5, indicating that the majority required only low-to-moderate–intensity maintenance therapy. Importantly, no patients in this cohort required dupilumab as part of their primary asthma treatment strategy.

### Treatment phases and study design

The study protocol was structured into 3 defined treatment phases, as illustrated in Fig 1, *B.* Term 1 was defined as the period from dupilumab initiation to 3 months, during which all patients received 300 mg dupilumab every 2 weeks while continuing PSL at about 4.5 mg/d. This period reflects the early clinical response induced by dupilumab treatment without any changes in concomitant therapy. Term 2 extended from month 3 to year 1 and represented the phase of PSL tapering, during which the dupilumab dosing interval was maintained at once every 2 weeks. This period aimed to assess the clinical impact of corticosteroid reduction under stable biologic treatment. Term 3 spanned from year 1 to year 3, during which PSL was either discontinued or maintained at minimal doses (≤1 mg), and the dupilumab dosing interval was extended to ≥3 weeks (average, 4 weeks). This phase was specifically intended to evaluate the influence of dosing interval extension on both upper and lower airway disease control. These 3 distinct treatment phases formed the basis for longitudinal evaluation of clinical outcomes.

### Clinical assessments

Clinical data were retrospectively collected from medical records at 5 time points: baseline (before treatment), then 3 months, 1 year, 2 years, and 3 years after the initiation of dupilumab therapy.

Decisions regarding PSL dose reduction and subsequent extension of the dupilumab dosing interval were made with careful clinical judgment that was based on consistently stable asthma control during the preceding 3 months. These treatment modifications were implemented only after obtaining informed consent from each patient and confirming that no exacerbation or deterioration in respiratory function had occurred.

Upper airway outcomes included NPS, computed tomography (CT) score, olfactory function, and rhinomanometry (inspiratory phase). NPS was assessed via nasal endoscopy and scored from 0 to 4 for each nasal cavity (total score, 0-8), with higher scores indicating more extensive polyposis.^10^ CT findings were classified using the standard Lund-Mackay grading system.^11^ The Self-Administered Odor Questionnaire (SAOQ)^12^ and the recognitive threshold score of Toyoda and Takagi (T&T) olfactometry^13^ were used to evaluate subjective and objective olfactory function, respectively.

Lower airway outcomes included spirometry (FEV_1_, percentage predicted FEV_1_ [%FEV_1_]), forced oscillation technique (R5, R5-20, Fres), fractional exhaled nitric oxide (Feno), peripheral blood eosinophil count, serum total IgE, and patient-reported asthma symptoms. The ACT was used to assess asthma control, consisting of 5 items scored from 1 to 5, with a total score ranging from 5 to 25. Higher ACT scores indicate better asthma control, and a score of ≥20 is considered to indicate well-controlled disease.^12^ Lung function was measured using a spirometer (HI-801, CHEST M.I., Tokyo, Japan), and Feno was measured with NO Breath (Bedfont Scientific, Maidstone, Kent, England, United Kingdom) per American Thoracic Society/ European Respiratory Society recommendations.^14^ Respiratory impedance was evaluated with the MostGraph-02 system (CHEST M.I.) using tidal breathing.

Asthma treatment steps were classified according to the 2024 Global Initiative for Asthma (GINA) strategy report.^7^ Treatment intensity was recorded at each time point, and patients were stratified for subgroup analysis according to whether step-down occurred during follow-up.

### Statistical analysis

Statistical analyses were conducted by GraphPad Prism v10 (GraphPad Software, La Jolla, Calif). The Shapiro-Wilk test was used to assess normality of data distribution. Paired comparisons between time points were performed by paired *t* tests for normally distributed variables and Wilcoxon matched-pairs signed-rank tests for nonparametric data. Continuous variables were reported as means ± SDs or medians with interquartile range as appropriate. All *P* values were 2 tailed, and *P* < .05 was considered statistically significant.

## Results

**Fig 2:**
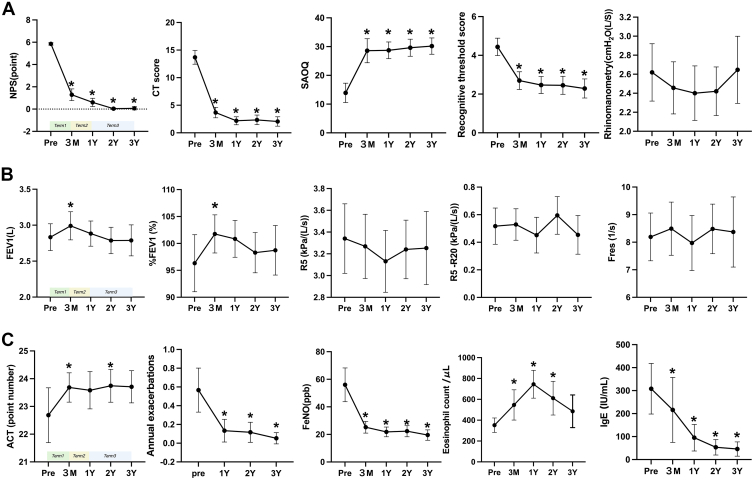
Longitudinal changes in upper and lower airway clinical parameters during dupilumab treatment. **A,** Upper airway outcomes include NPS, Lund-Mackay CT score, Self-Administered Odor Questionnaire (SAOQ), T&T olfactometry, and rhinomanometry. **B,** Lower airway outcomes include spirometry (FEV_1_ and %FEV_1_) and forced oscillation technique parameters (R5, R5-20, Fres). **C,** Asthma-related outcomes include ACT scores, annual asthma exacerbation frequency, Feno, peripheral blood eosinophil count, and total serum IgE. Error bars represent 95% confidence intervals (CIs). Statistical significance (*P* < .05) vs baseline (Pre) is denoted by asterisks.

Time-course changes in upper airway, pulmonary function, and asthma-related inflammatory and clinical control markers were evaluated. Fig 2, *A,* shows the evolution of upper airway parameters. NPS, CT score, olfactory function measured by T&T olfactometry, and the Self-Administered Odor Questionnaire all demonstrated marked improvement within term 1, suggesting a rapid therapeutic response to dupilumab. These improvements were sustained throughout term 2 and term 3, with no indication of rebound disease. Rhinomanometry values also improved during term 1 but showed a trend toward worsening during term 3, after extension of the dupilumab dosing interval. However, these changes did not reach statistical significance. Fig 2, *B,* illustrates pulmonary function outcomes. FEV_1_ and %FEV_1_ both increased significantly during term 1. Subsequently, these values gradually declined during term 2 and term 3 and approached baseline by year 3.

Forced oscillation technique parameter R5 followed a similar trajectory, with transient improvement during term 1 and mild deterioration during term 3. In contrast, R5-20 and Fres remained relatively unchanged across all treatment phases, showing no consistent trends in response to dupilumab therapy, PSL tapering, or dosing interval extension. Fig 2, *C,* shows asthma control and inflammation-related biomarkers. ACT scores improved significantly in term 1 and remained stably elevated during term 2 and term 3. The number of annual asthma exacerbation frequency decreased substantially after dupilumab initiation and remained suppressed over the full 3-year observation period. Feno also declined during term 1 and stayed low thereafter. Peripheral blood eosinophil counts showed a transient increase during term 1 and a further rise during term 2, consistent with corticosteroid tapering. Peripheral blood eosinophil counts increased during term 2 in association with corticosteroid tapering, but subsequently declined during term 3 after the extension of dupilumab dosing intervals, and remained slightly above baseline levels. Total serum IgE levels demonstrated a steady and progressive decline throughout the study period.

**Fig 3:**
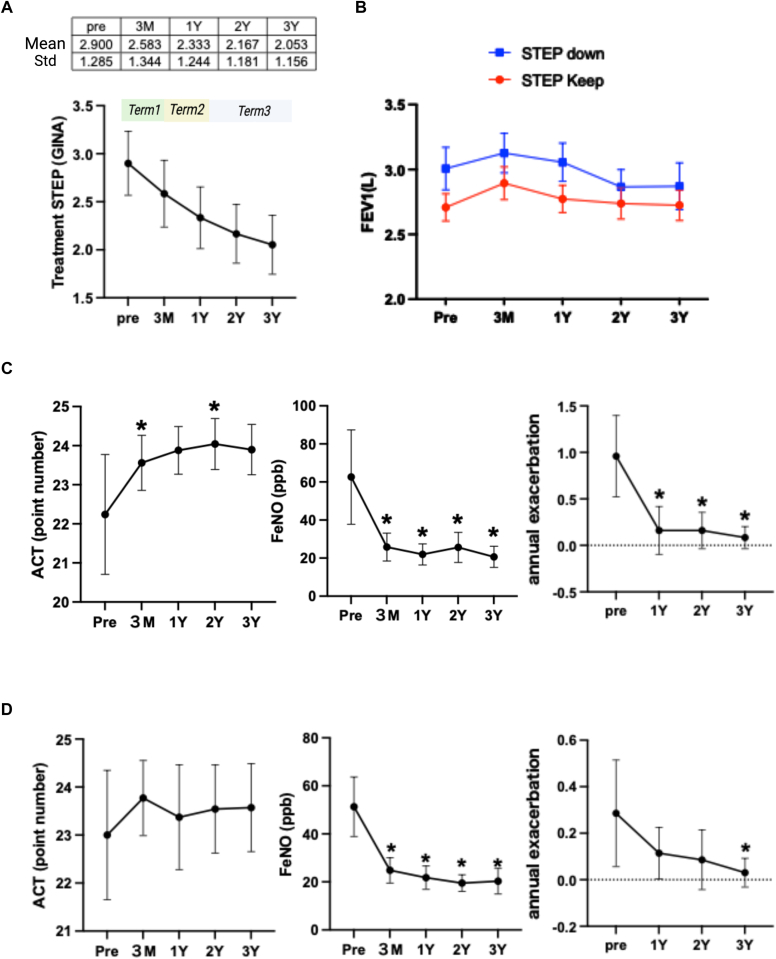
**A,** Mean asthma treatment step level based on GINA classification across 3-year observation period. **B,** Comparison of FEV_1_ between patients with and without asthma treatment step-down. **C,** Longitudinal changes in ACT, Feno, and annual exacerbation frequency in step-down group. **D,** Longitudinal changes in ACT, Feno, and annual exacerbation frequency in non–step-down group. Statistical significance (*P* < .05) vs baseline (Pre) is denoted by asterisks.

Asthma treatment was gradually stepped down in the majority of patients (Fig 3, *A*). The mean GINA step decreased from 2.9 at baseline to 2.05 at year 3, suggesting that asthma control was maintained despite PSL tapering and extension of dupilumab dosing intervals.

To assess the impact of treatment de-escalation, patients were stratified into two subgroups according to whether they underwent a step-down in asthma medication during the study period. At baseline, the mean asthma treatment step was significantly higher in the step-down group (3.80) than in the non–step-down group (2.25) (*P* < .0001), indicating that step-down occurred primarily in patients with initially more intensive therapy (data not shown). Among those who underwent step-down, FEV_1_ values were generally higher compared to those who maintained baseline treatment intensity. Although a modest decline in FEV_1_ was observed during term 3, the overall trend remained consistent with that of the entire cohort (Fig 3, *B*). In addition, ACT scores, Feno levels, and annual asthma exacerbation frequency in the step-down group followed trajectories similar to those observed in the full population. These parameters showed significant improvement during term 1 and remained stable throughout term 2 and term 3, suggesting that dupilumab enabled a safe reduction in asthma treatment intensity without loss of disease control (Fig 3, *C*).

Furthermore, Fig 3, *D,* presents data from patients who did not undergo asthma treatment step-down. In this subgroup, ACT scores were already within the well-controlled range at baseline and remained consistently high throughout the 3-year follow up. Similarly, Feno levels and annual exacerbation frequency demonstrated stable or improving trends comparable to those seen in the step-down group. These findings suggest that asthma control was successfully maintained over time, regardless of whether treatment de-escalation was pursued.

**Fig 4:**
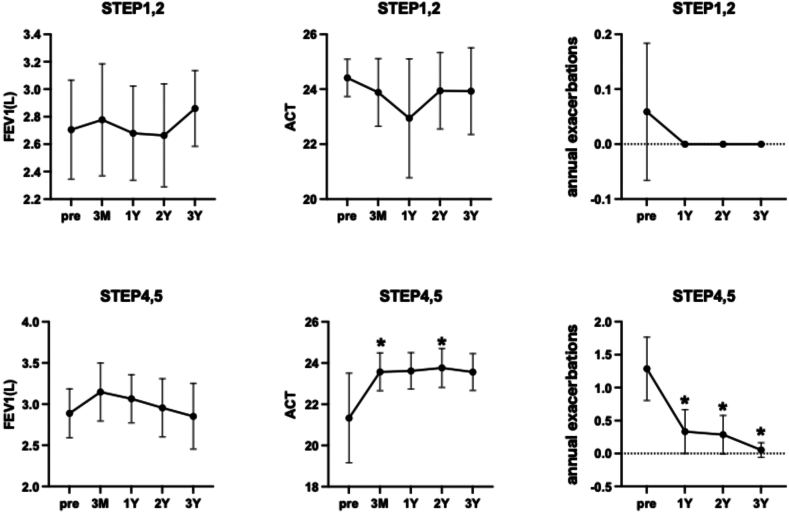
Subgroup analysis stratified by initial asthma severity. Patients were divided into those with mild asthma (GINA steps 1-2) and those with severe asthma (GINA steps 4-5). Asthma-related outcomes including FEV_1_, ACT, and exacerbation frequency are presented over time. Error bars represent 95% confidence intervals (CIs). Statistical significance (*P* < .05) vs baseline (Pre) is denoted by asterisks.

Given that this study targeted patients with recurrent eosinophilic CRSwNP, the cohort included individuals with varying degrees of asthma severity. Patients were stratified into mild asthma (GINA steps 1-2) and severe asthma (GINA steps 4-5) subgroups. In the mild asthma group, none of the evaluated parameters, including FEV_1_, ACT, Feno, and exacerbation rates, demonstrated significant changes throughout the study, indicating stable disease. In contrast, patients with severe asthma showed clinically meaningful improvements in ACT and exacerbation frequency during term 1. These improvements were sustained during term 2 and term 3, even with PSL tapering and dupilumab dosing interval extension (Fig 4).

## Discussion

This study demonstrates that in patients with CRSwNP and comorbid asthma who obtained good control of sinonasal disease, tapering of both systemic corticosteroids and dupilumab dosing intervals was feasible without loss of asthma control, supporting the safety of a stepwise de-escalation protocol under stable upper airway conditions. By evaluating both upper and lower airway parameters longitudinally, we provide robust real-world evidence supporting a tapering strategy that aligns with patient-centered care goals and treatment de-escalation.

The concept of *united airway disease* posits a shared pathophysiologic basis for upper and lower airway disorders, particularly type 2 inflammation mediated by IL-4, IL-5, and IL-13.^1^^,^^2^ Mechanistic insights from recent reports underscore the multilevel interactions between the upper and lower airways. Neural regulation via pathways such as the nasobronchial reflex,^15^ along with common inflammatory mediators including IL-33, IL-25, and thymic stromal lymphopoietin,^16^ enable bidirectional immune signaling across the airway axis. Furthermore, emerging evidence suggests that extracellular vesicles, particularly exosomes released from epithelial and immune cells, facilitate interairway communication and modulate type 2 inflammation.^17^^,^^18^ Airway microbiota may contribute to the persistence of chronic T_H_2-type inflammation across both the upper and lower airways not only through direct postnasal drainage but also via endotoxin-mediated inflammatory signaling; however, the precise mechanisms underlying these interactions remain unclear.^19^^,^^20^ These multifaceted mechanisms support a unified therapeutic strategy and emphasize the potential for biologics to exert pan-airway effects.^1^^,^^2^^,^^21^ Consistent with this concept, clinical studies have shown that disease of patients with asthma and comorbid eosinophilic CRSwNP is more likely to exhibit superresponse status and obtain remission with biologics compared to patients with asthma alone.^22^

Our findings resonate with this framework. Improvement in NPS, olfactory function, and radiologic findings were observed within the first 3 months of dupilumab therapy (term 1), coinciding with early improvements in ACT scores and reductions in Feno and exacerbation rates. These improvements were sustained through systemic corticosteroid tapering and biologic interval extension (terms 2 and 3), suggesting that upper airway control may beneficially influence lower airway inflammation, as hypothesized by the UAD model.

Notably, while FEV_1_ and R5 showed modest declines during term 3, no corresponding deterioration was observed in symptoms, Feno, or exacerbation rates. This dissociation between spirometric and clinical outcomes is consistent with previous reports indicating that patients with mild airflow limitation often do not perceive symptoms despite measurable reductions in lung function.^23^ In our cohort, baseline lung function was relatively preserved (mean FEV_1_, 2.83 L), and the proportion of patients in GINA steps 1, 2, and 3 was high. Compared to previous phase 2 and 3 trials that enrolled patients with uncontrolled severe asthma and lower average FEV_1_ values (1.58-1.84 L),^24^^,^^25^ our cohort likely had less symptomatic sensitivity to small functional declines. These observations highlight the need to interpret physiologic and clinical asthma measures in tandem, particularly in biologic-treated patients with comorbid CRSwNP.

Furthermore, the immunologic durability of dupilumab, as evidenced by sustained reductions in total serum IgE and Feno, likely contributes to long-term disease control even with reduced treatment intensity. These observations are consistent with findings from both randomized controlled trials and real-world studies of dupilumab, which have demonstrated sustained suppression of type 2 inflammatory biomarkers and long-term clinical benefit under standard dosing regimen of administration every 2 weeks.^26^, ^27^, ^28^, ^29^ Remarkably, our results show that even with extension of the dupilumab dosing interval to ≥3 weeks, suppression of IgE and Feno was comparable to these prior studies, suggesting that the immunomodulatory effects of dupilumab may persist despite reduced dosing frequency. This persistent immunologic control may explain the safe implementation of asthma treatment step-down observed in the majority of patients over 3 years.

**Supplementary Fig E1:**
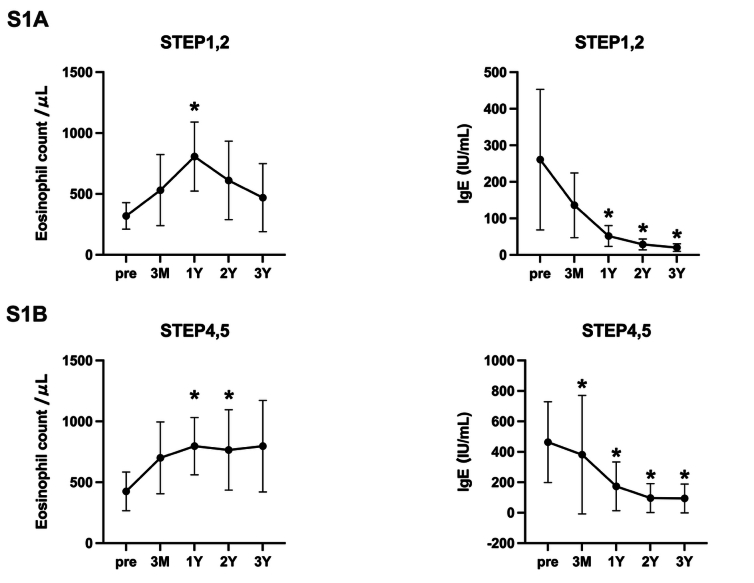


Subgroup analysis showed that patients with severe asthma (GINA steps 4-5) experienced early improvements in ACT scores and exacerbation frequency after dupilumab initiation, with sustained benefits during corticosteroid tapering and dosing interval extension. While a prior phase 2b trial reported reduced efficacy with 4-week dupilumab dosing in patients with uncontrolled asthma,^25^ our findings should not be interpreted as supporting interval extension in patients with severe asthma alone. All patients in our cohort had comorbid CRSwNP and relatively well-controlled asthma at baseline—key differences from the trial population, in which only 16% had CRSwNP.^25^ In step 4-5 patients, serum IgE levels declined after interval extension, whereas peripheral eosinophil counts remained elevated (see Fig E1, *B,* in the Online Repository available at www.jaci-global.org). Despite this, asthma control was maintained, consistent with prior reports suggesting that dupilumab-induced increases in blood eosinophils result from reduced tissue migration, not increased disease activity.^30^ In contrast, step 1-2 patients showed little clinical change, likely as a result of a ceiling effect from good baseline asthma control. Notably, their IgE and eosinophil levels improved in parallel with the overall cohort, indicating preserved biological responsiveness despite minimal clinical shifts (Fig E1, *A*).

Step-down in asthma pharmacotherapy was obtained in most patients without loss of disease control. The average GINA step decreased from 2.9 at baseline to 2.05 by year 3, with stable ACT scores, Feno levels, and exacerbation frequency, supporting the feasibility of de-escalation under appropriate monitoring. Reduction of inhaled asthma therapy was undertaken only after confirming stable asthma control for at least 3 months and obtaining informed consent. While real-world evidence remains limited, some studies have reported decreased receipt of inhaled corticosteroids during dupilumab treatment.^31^ Our findings further demonstrate that such de-escalation is feasible even with extended dupilumab dosing intervals (≥3 weeks). This highlights the potential to reduce both systemic and biologic treatment intensity in selected patients with well-controlled upper and lower airway disease.

This study’s strengths include comprehensive longitudinal assessment, real-world setting, and integration of both subjective and objective airway measures. The inclusion of dosing interval changes adds practical relevance for clinicians aiming to reduce treatment burden. Limitations include the absence of a control group and the observational design, which limits causal inference. Our cohort included only CRSwNP patients with adequate disease control to permit PSL tapering and dupilumab interval extension, which may bias results toward patients with responsive disease. Moreover, patients with severe asthma alone were not included, as our center primarily serves CRSwNP surgical cases. Thus, direct comparison with asthma-only populations was not feasible, and findings should be interpreted accordingly. However, because enrollment was not restricted by asthma severity or treatment response, selection bias within the asthma subgroup is unlikely. The consistent trends observed across subgroups support the internal validity of our findings.

In summary, this study demonstrates that in patients with CRSwNP and comorbid asthma, once sinonasal inflammation is well controlled with dupilumab, both PSL tapering and dosing interval extension can be implemented without compromising lower airway outcomes. These findings support the unified airway model, suggesting that effective sinonasal management contributes to sustained asthma control, even as treatment intensity is reduced. Importantly, the feasibility of extending dosing intervals may have additional relevance in resource-limited settings where the high cost of biologics poses a barrier to long-term access. Strategic de-escalation guided by disease control may thus offer a cost-conscious approach to optimizing care. Further prospective studies are needed to validate these observations and refine long-term treatment strategies.

**Key messages**

• Dupilumab effectively improves both upper and lower airway outcomes in patients with CRSwNP and comorbid asthma.
• Prednisolone tapering and extension of dupilumab dosing intervals can be safely obtained without loss of asthma control when sinonasal disease is well controlled.
• These findings support the feasibility of treatment de-escalation strategies in the context of UAD.

## Disclosure statement

Disclosure of potential conflict of interest: The authors declare that they have no relevant conflicts of interest.

## References

[1] Giavina-Bianchi P., Vivolo Aun M., Takejima P., Kalil J., Agondi R.C. (2016). United airway disease: current perspectives. J Asthma Allergy.

[2] Kanda A., Kobayashi Y., Asako M., Tomoda K., Kawauchi H., Iwai H. (2019). Regulation of interaction between the upper and lower airways in united airway disease. Med Sci.

[3] Maspero J.F., Katelaris C.H., Busse W.W., Castro M., Corren J., Chipps B.E. (2020). Dupilumab efficacy in uncontrolled, moderate-to-severe asthma with self-reported chronic rhinosinusitis. J Allergy Clin Immunol Pract.

[4] Bachert C., Han J.K., Desrosiers M., Hellings P.W., Amin N., Lee S.E. (2019). Efficacy and safety of dupilumab in patients with severe chronic rhinosinusitis with nasal polyps (LIBERTY NP SINUS-24 and LIBERTY NP SINUS-52): results from two multicentre, randomised, double-blind, placebo-controlled, parallel-group phase 3 trials. Lancet.

[5] Fokkens W.J., Lund V.J., Hopkins C., Hellings P.W., Kern R., Reitsma S. (2020). European position paper on rhinosinusitis and nasal polyps 2020. Rhinology.

[6] Nakashima D., Nakayama T., Minagawa S., Adachi T., Mitsuyama C., Shida Y. (2025). Effectiveness of dupilumab treatment against refractory eosinophilic chronic rhinosinusitis. J Allergy Clin Immunol Glob.

[7] Global Initiative for Asthma (GINA) (2024). Global strategy for asthma management and prevention. https://ginasthma.org/archived-reports/.

[8] van der Lans R.J.L., Otten J.J., Adriaensen G.F.J.P.M., Hoven D.R., Benoist L.B., Fokkens W.J. (2023). Two-year results of tapered dupilumab for CRSwNP demonstrates enduring efficacy established in the first 6 months. Allergy.

[9] Yoon S.Y., Cha H., Hong S.N., Yang M.S., Kim D.W. (2024). Therapeutic effectiveness of SNOT 22–based interdose interval adjustment of dupilumab for chronic rhinosinusitis with nasal polyps. Clin Exp Otorhinolaryngol.

[10] Tsetsos N., Goudakos J.K., Daskalakis D., Konstantinidis I., Markou K. (2018). Monoclonal antibodies for the treatment of chronic rhinosinusitis with nasal polyposis: a systematic review. Rhinology.

[11] Lund V.J., Kennedy D.W. (1997). Staging for rhinosinusitis. Otolaryngol Head Neck Surg.

[12] Takebayashi H., Tsuzuki K., Oka H., Fukazawa K., Daimon T., Sakagami M. (2011). Clinical availability of a self-administered odor questionnaire for patients with olfactory disorders. Auris Nasus Larynx.

[13] Miwa T., Ikeda K., Ishibashi T., Kobayashi M., Kondo K., Matsuwaki Y. (2019). Clinical practice guidelines for the management of olfactory dysfunction—secondary publication. Auris Nasus Larynx.

[14] Dweik R.A., Boggs P.B., Erzurum S.C., Irvin C.G., Leigh M.W., Lundberg J.O. (2011). An official ATS clinical practice guideline: interpretation of exhaled nitric oxide levels (Feno) for clinical applications. Am J Respir Crit Care Med.

[15] Hens G., Raap U., Vanoirbeek J., Meyts I., Callebaut I., Verbinnen B. (2011). Selective nasal allergen provocation induces substance P–mediated bronchial hyperresponsiveness. Am J Respir Cell Mol Biol.

[16] Hong H., Liao S., Chen F., Yang Q., Wang D.Y. (2020). Role of IL-25, IL-33, and TSLP in triggering united airway diseases toward type 2 inflammation. Allergy.

[17] Zheng Z., Yu Y. (2022). A review of recent advances in exosomes and allergic rhinitis. Front Pharmacol.

[18] Zhang W., Zhang J., Cheng L., Ni H., You B., Shan Y. (2018). A disintegrin and metalloprotease 10–containing exosomes derived from nasal polyps promote angiogenesis and vascular permeability. Mol Med Rep.

[19] Zhou Y., Jackson D., Bacharier L.B., Mauger D., Boushey H., Castro M. (2019). The upper-airway microbiota and loss of asthma control among asthmatic children. Nat Commun.

[20] Kim Y.C., Won H.K., Lee J.W., Sohn K.H., Kim M.H., Kim T.B. (2019). *Staphylococcus aureus* nasal colonization and asthma in adults: systematic review and meta-analysis. J Allergy Clin Immunol Pract.

[21] Domínguez-Ortega J., Mullol J., Álvarez Gutiérrez F.J., Miguel-Blanco C., Castillo J.A., Olaguibel J.M. (2024). The effect of biologics in lung function and quality of life of patients with united airways disease: a systematic review. J Allergy Clin Immunol Glob.

[22] Hamada S., Ogino E., Yasuba H. (2025). Eosinophilic chronic rhinosinusitis as a predictor of super-responder and clinical remission in patients with uncontrolled type 2–high severe asthma treated with biologics. Respir Investig.

[23] Boulay M.E., Boulet L.P. (2013). Discordance between asthma control clinical, physiological and inflammatory parameters in mild asthma. Respir Med.

[24] Castro M., Corren J., Pavord I.D., Maspero J., Wenzel S., Rabe K.F. (2018). Dupilumab efficacy and safety in moderate-to-severe uncontrolled asthma. N Engl J Med.

[25] Wenzel S., Castro M., Corren J., Maspero J., Wang L., Zhang B. (2016). Dupilumab efficacy and safety in adults with uncontrolled persistent asthma despite use of medium-to-high-dose inhaled corticosteroids plus a long-acting β2 agonist: a randomised double-blind placebo-controlled pivotal phase 2b dose-ranging trial. Lancet.

[26] Rabe K.F., Nair P., Brusselle G., Maspero J.F., Castro M., Sher L. (2018). Efficacy and safety of dupilumab in glucocorticoid-dependent severe asthma. N Engl J Med.

[27] Wechsler M.E., Ford L.B., Maspero J.F., Pavord I.D., Papi A., Bourdin A. (2022). Long-term safety and efficacy of dupilumab in patients with moderate-to-severe asthma (TRAVERSE): an open-label extension study. Lancet Respir Med.

[28] Laidlaw T.M., Bachert C., Amin N., Desrosiers M., Hellings P.W., Mullol J. (2021). Dupilumab improves upper and lower airway disease control in chronic rhinosinusitis with nasal polyps and asthma. Ann Allergy Asthma Immunol.

[29] Numata T., Araya J., Miyagawa H., Okuda K., Takekoshi D. (2022). Real-world effectiveness of dupilumab for patients with severe asthma: a retrospective study. J Asthma Allergy.

[30] Portacci A., Poto R., Varricchi G., Carpagnano G.E. (2025).

[31] Faverio P., Ronco R., Monzio Compagnoni M., Franchi M., Franco G., Bonaiti G. (2023). Effectiveness and economic impact of dupilumab in asthma: a population-based cohort study. Respir Res.

